# Socio-ecological risk factors associated with different health-risk behaviors among Chinese adolescents: a cross-sectional study

**DOI:** 10.3389/fpubh.2026.1735957

**Published:** 2026-10-27

**Authors:** Xian Wang, Wanting Chao, Xili Jiang, Xiaomin Lou, Rui Ma

**Affiliations:** 1 College of Public Health, Zhengzhou University, Henan, Zhengzhou, China; 2 School of Kinesiology and Physical Education, Zhengzhou University, Henan, Zhengzhou, China

**Keywords:** adolescents, health risk behaviors, prevention, public health, socio-ecological risk

## Abstract

**Objective:**

To explore the association between the level of socio-ecological risk factors and adolescent health risk behaviors, and to provide theoretical basis for reducing the occurrence of adolescent risk behaviors and improving adolescent behavioral problems.

**Methods:**

This cross-sectional study was conducted in Zhengzhou City and 3377 participants were enrolled. Perceived socio-ecological risk was measured using a validated 50-item questionnaire. Grounded in ecological systems theory, investigating the risk factors associated with adolescent health risk behaviors across individual, family, school, community, policy, cultural and chronosystem level. Health risk behavior outcomes including diet, physical activity, sleep duration and screen time on weekdays and weekends, drinking and smoking behaviors and then divided into healthy and unhealthy groups. Independent t-test, ANOVA and chi-square test were applied for descriptive group comparisons. Binary logistic regression was performed to estimate associations between multi-level perceived socio-ecological risk and health risk behaviors. Demographic covariates covered gender, only-child status, residential areas, learning period, parental education, family economic status, number of friends and study burden.

**Results:**

After adjusting for confounding factors, compared with participants in the low-risk group for total socio-ecological risk, participants in the high-risk group showed positive association between irregular diet (*OR*: 4.021, 95% *CI*: 3.145–5.141), lack of physical exercise (*OR*: 1.779, 95% *CI*: 1.400–2.262), short sleep time (weekday: *OR*: 1.475, 95% *CI*: 1.059–2.056, weekend: *OR*: 1.287, 95% *CI*: 1.048–1.580), long screen time (weekday: *OR*: 1.399, 95% *CI*: 1.090–1.797, weekend: *OR*: 2.086, 95% *CI*: 1.678–2.592), smoking (*OR*: 3.964, 95% *CI*: 2.894–5.428) and drinking (*OR*: 3.662, 95% *CI*: 2.321–5.778) and high social-ecological risk. The higher the risk of social-ecological risk, the higher the risk of developing health risk behaviors.

**Conclusion:**

The results supported the importance of investigating health risk behaviors in adolescents and the health risk behaviors were associated with socio-ecological risk

## 1 Introduction

The World Health Organization (WHO) defines adolescent health-risk behaviors as actions that directly or indirectly compromise adolescents’ present and future health, including tobacco use, alcohol consumption, suicidal and self-harm behaviors, unhealthy dietary patterns, and insufficient physical activity (1). The emergence of a series of adolescent health problems is not represented by intuitive indicators such as prevalence or mortality, but is more closely related to behavior (2). Adolescence represents a critical transitional period of physical, psychological, and social maturation, during which long-term health-related behaviors are shaped (3). As self-awareness grows, teenagers in this period desire to pursue independence and are eager to break free from parental supervision (4). Limited risk awareness renders them susceptible to behavioral deviations without timely detection and appropriate guidance (5). With age increasing, the prevalence of health risk behaviors increases, these behaviors tend to exhibit clustering or co-occurrence and may persist into adulthood to produce lifelong health impacts.

Global epidemiological evidence has suggested a high burden of multiple health-risk behaviors among adolescents worldwide: insufficient physical activity (6), excessive screen-time (7), insufficient sleep (8), unhealthy diet (9), tobacco use (10) and alcohol drinking (11) are highly prevalent and predispose individuals to long-term non-communicable disease risk (12). Although some unhealthy behaviors have been studied, fewer studies have systematically explored how multi-level environmental background jointly drives these multiple risk behaviors among Chinese adolescents. Given the serious consequences of health risk behaviors developed during adolescence, this study aims to clarify socio-ecological risk factors that may influence health risk behaviors among adolescents.

From a socio-ecological perspective, individuals exist within a layered structure of interconnected environmental systems, ranging from personal and familial to societal and cultural contexts (13, 14). The socio-ecological model constitutes a foundational theoretical framework in public health, as it highlights how environmental characteristics shape individual health behaviors and related outcomes (15). Despite these theoretical advances, existing empirical work applying the socio-ecological model to adolescent health-risk behaviors has notable limitations. Most prior investigations focus on only one or a small subset of risk behaviors and examine limited socio-ecological domains, which cannot capture the multidimensional nature of real world adverse exposures experienced by adolescents (16).

Accordingly, the primary objective of this study is to examine the associations between the multi-dimensional factors of socio-ecological risk (individual, family, school, community, policy, culture, and chronosystem) and various health risk behaviors (diet, physical activities, sleep duration on weekdays and weekends, screen time on weekdays and weekends, drinking, and smoking) during adolescence. This study further intends to build a comprehensive multi-domain socio-ecological analytical framework for adolescent health-risk behaviors. The theoretical novelty lies in integrating seven socio-ecological dimensions to unpack how layered environmental risks correlate with multiple adolescent behavioral problems. Practically, it generates evidence in the development of ecosystem-based health behavior models that are relevant for efforts to prevent health risk behavior in adolescents.

## 2 Methods

### 2.1 Study population

The convenient sampling design was used in this cross-sectional survey targeting adolescents. Firstly, two middle schools, four high schools and one general university were selected where the questionnaire survey was administered. Subsequently, all the students from the randomly selected classes from these grades were included in this research. Approximately 200 students were selected in each grade. In general, a total of 4351 students were investigated, and participants with missing data on the items of socio-ecological risk (*n* = 366) were excluded. Additionally, students provided incomplete responses on health risk behaviors (*n* = 330) or demographic characteristics (*n* = 220), and some abnormal data (*n* = 58) were eliminated. Finally, a sample of 3377 participants was obtained (Figure 1). Sensitivity analysis showed no statistical differences in the distribution between the original data and the final analysis data in terms of gender, family residence, parental education level, self-reported family economy, close friends, and study burden (Supplementary Table A1).

**Figure 1:**
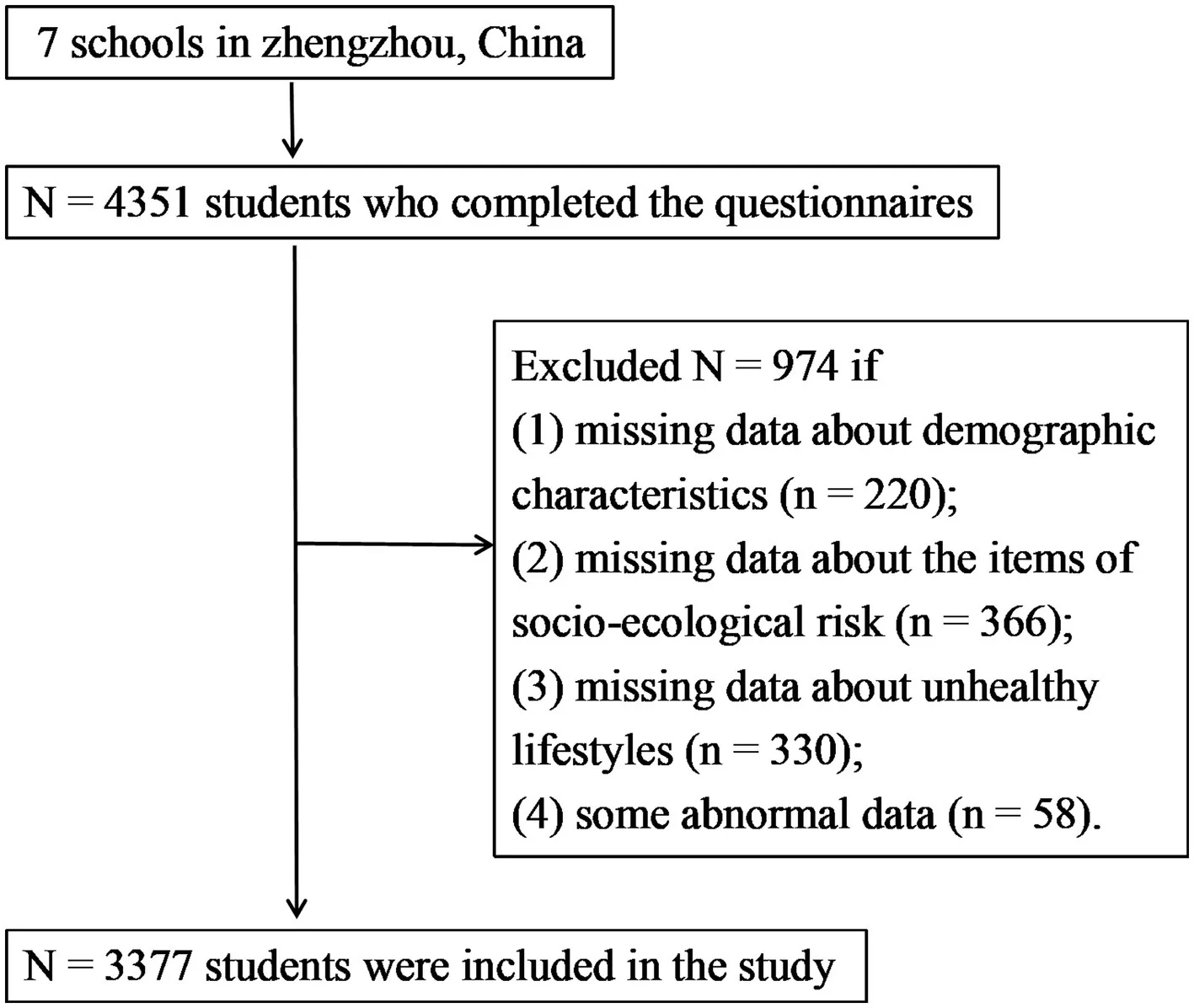
Flowchart of the study.

For this study, the protocol was approved by the Zhengzhou University Life Science Ethics Committee (Code: ZZUIRB2021-94). A self-administered anonymous survey was conducted with the support of well-trained data collectors. Prior to commencing the research, all participants and their guardians were required to sign the informed consent.

### 2.2 Measures

The exposure to social ecological risk was assessed with the Social Ecological Risk Assessment Questionnaire of Adolescents involving seven dimensions (individual, family, school, community, policy, culture, and chronosystem). The 50-item questionnaire was designed by Chinese scholars Tao et al. and has been verified in the Chinese adolescent population (17, 18). All items were scored using Likert’s 5-point scale (Supplementary Table A2) and items 4, 7, 8, 14–16, 24–33, 40–50 were reverse items, which were transformed into the same direction as the other items during the analysis. Each item was set with 5 options: 1 = completely consistent, 2 = basically consistent, 3 = somewhat consistent, 4 = basically inconsistent and 5 = very inconsistent. The total score of cumulative ecological risk exposure is the sum of the scores of all items. The higher the score, the higher the level of cumulative ecological risk exposure. The *Cronbach’s alpha* coefficient was 0.893 and the *KMO* value was 0.908 (Supplementary Table A2). According to the threshold values of *P_25_* and *P_75_*, the exposure levels of each dimension were divided into three levels, where ≤ *P_25_* represented low level of risk, > *P_25_* and ≤ *P_75_* represented medium level of risk, and > *P_75_* represented high level of risk (17, 18).

This research on health risk behaviors of adolescents included six aspects: diet (7 items), physical activities (PA, 3 items), sleep duration (2 items), screen time (ST, 2 items), drinking (1 item), and smoking (1 item). Seven items of diet included sugar-sweetened beverages, soda beverages, fast food, breakfast, fruits, vegetables, and dried fruit. Total score of the seven items was summed up, and the median total score was calculated (median = 4). The total score of diet was classified as healthy (< 4) and unhealthy (≥ 4) (19). PA was scored by the Physical Activity Registration Scale, amount of PA = time × frequency × intensity. Intensity and frequency were rated on a scale of 1–5 and time was assigned from 0 to 4. The amount of PA ranged from 0–100, which was classified as unhealthy (≤ 19) and healthy (> 19) (20). Sleep duration was divided into two variables: weekday and weekend sleep duration. The method of calculating sleep duration: Sleep duration = Time to wake up - Time to go to bed - Time to fall asleep. Sleep duration was classified as unhealthy (junior high school: < 9 h per day; high school and college: < 8 h per day) and healthy (junior high school: ≥ 9 h per day; high school and college: ≥ 8 h per day) (21). Adolescents’ daily ST was also divided into two variables: weekday and weekend ST. ST was classified as healthy (< 2 h per day) and unhealthy (≥ 2 h per day) (22). Regarding drinking, it was classified as unhealthy (having drinking behavior) and healthy (not having drinking behavior during the past a moth). Smoking was dealt with in the same way as drinking (23). Additionally, participants were asked to report several demographic data, including gender, only child status, residential areas, learning period, education levels of father and mother, self-assessed family economy, number of friends and study burden.

### 2.3 Data analysis

SPSS 27.0 was used for data analysis. First, independent sample T-test and ANOVA test were used to observe differences of socio-ecological risk scores among demographic characteristics. Secondly, the chi-square test was used to test differences in the percentages of health risk behaviors among demographic variables and the groups of low, moderate and high risk from each dimension and the total score of socio-ecological risk. In the last step, binary logistic regression estimated the association between the proportions of health risk behaviors and the groups of low, moderate and high risk from each dimension and total score of socio-ecological risk. In the crude model (Model 1), only socio-ecological risk factors were incorporated, whereas all other covariates (gender, only child status, residential areas, learning period, the education level of father and mother, self-assessed family economy, number of friends and study burden) were additionally included in the adjusted model (Model 2). Bilateral *p* < 0.05 was considered statistically significant in the analyses.

## 3 Results

### 3.1 Comparison of socio-ecological risk scores among different demographic characteristics

Demographic subgroups showed measurable differences across multiple socio-ecological risk dimensions (Supplementary Table A3). The risk scores of girls in the individual and cultural dimensions were higher than those of boys, while the risk scores of boys in the family, community and policy dimensions were higher than those of girls (*p* < 0.05). Urban–rural differences were also detected, urban adolescents obtained higher scores for the cultural and chronosystem dimensions, while rural adolescents showed higher risk scores for other dimensions (*p* < 0.05). The risk scores showed significant differences across different educational stages, and each schooling group carried its own prominent high-risk dimension (*p* < 0.05). In addition, students with higher educational levels of their parents, better family economic conditions, more friends, and lighter academic burdens have lower social risks (*p* < 0.05).

### 3.2 Comparison of health risk behaviors with different demographic characteristics

As shown in Supplementary Table A4, females were healthier than males in terms of weekend sleep duration, smoking and drinking, but less healthy in terms of physical exercise and sleep time on weekdays. The proportion of students in rural areas who lack exercise and have long screen time was significantly higher than that of urban students. Students whose parents have a low educational level tend to have less physical activity and a higher rate of smoking. Students with more friends and lighter study burden have the lowest proportions of unhealthy diet and physical inactivity. Sleep insufficiency represented a substantial challenge for high-school students, and heavier academic burden went hand in hand with greater odds of inadequate sleep. Compared with weekdays, the proportion of screen usage time on weekends has increased, and the proportion of high school students has risen most significantly (from 5.7 to 61.3%). All group comparisons showed statistically significant differences.

### 3.3 The comparison of health risk behaviors among different socio-ecological risk groups

As shown in Figure 2 and Supplementary Table A5, for the total score of socio-ecological risk, the proportion of health risk behaviors in the medium-high risk group was higher than that in the low risk group, including irregular diet, lack of physical exercise, short sleep time, long screen time, smoking and drinking (*p* < 0.05). Similar results were also observed in the seven-dimensional risk score (including individual, family, school, community, policy, culture, and chronosystem), with higher risk scores associated with a higher proportion of health risk behaviors in adolescents.

**Figure 2:**
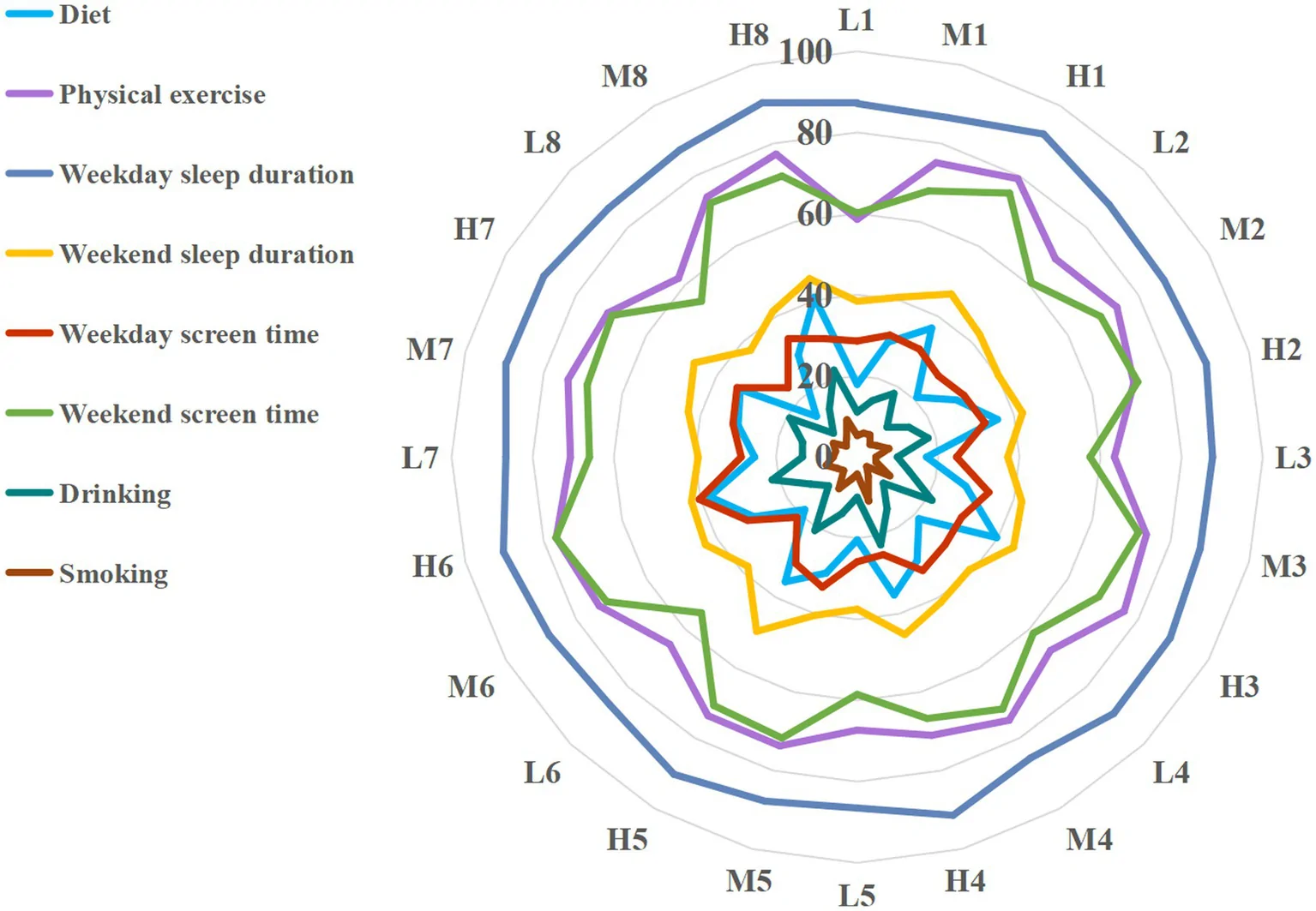
Unhealthy rates of various health risk behaviors at different levels of socio-ecological risk. Different colors in the first radar chart correspond to different health risk behaviors in the legend. The letters and numbers on the outer edge of the radar chart have the following meanings: L, M, and H indicate low, medium, and high score group. 1–7 represent each dimension and total score of socio-ecological risk: 1, Individual level; 2, Family level; 3, School level; 4, Community level; 5, Public policy; 6, Culture level; 7, Chronosystem; 8, Total risk score. For example, L1 represents low score group at the individual level. 0–100 in the radar chart represents the reference percentage for each row. For example, 37.5% indicates that the unhealthy rate of weekend sleep time is 37.5% for adolescents in the low group of policy (L5).

### 3.4 Associations between multi-level socio-ecological risk and health-risk behaviors

Adjustment for covariates did not eliminate the positive links between elevated total socio-ecological risk and each examined health-risk behavior (Supplementary Table A6 and Figure 3). Irregular diet stood out as the outcome with the strongest association against cumulative socio-ecological risk (*OR* = 4.021, 95% *CI*: 3.145–5.141). Four socio-ecological dimensions included the individual, family, policy and cultural levels. High-risk status in these domains consistently predicted higher odds for multiple health-risk behaviors even after confounding factors had been controlled. Individual, school, policy and cultural high-risk status each raised the probability of insufficient physical activity. High-risk status at policy and cultural levels related to shorter sleep duration regardless of whether observations fell on weekdays or weekends. High-risk exposure within individual, family and cultural dimensions contributed to longer screen time both on weekdays and weekends (*p* < 0.05).

**Figure 3:**
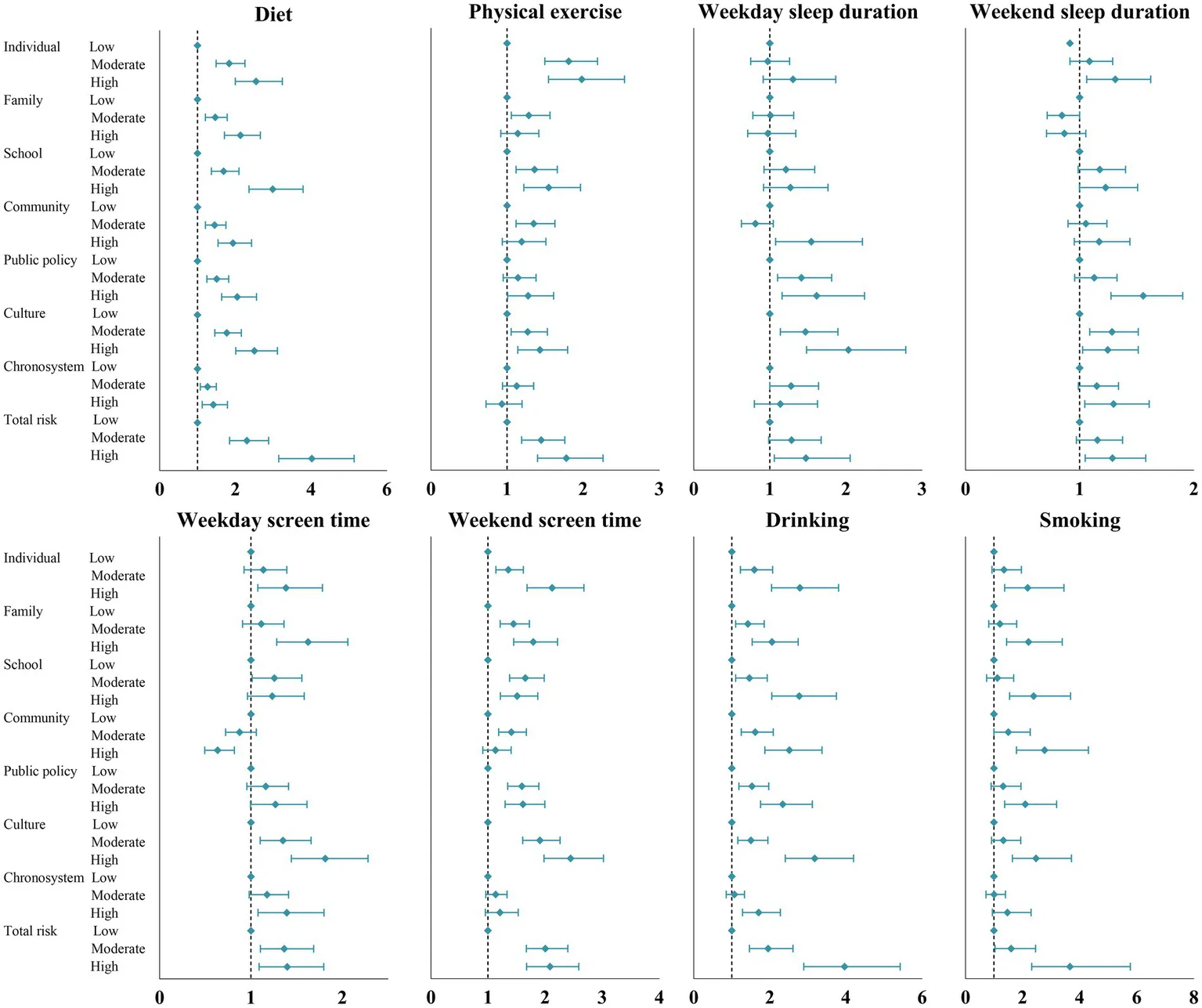
The associations between various health risk behaviors and socioecological risk. Model 1: crude model. Model 2 adjusted for gender, only child status, residential areas, learning period, the education level of father and mother, self-assessed family economy, number of friends and study burde.

In addition, it is noteworthy that in Model 1, compared with the low-risk group at the community level, the risk of long screen time for the high-risk group decreased by 25% on weekdays (*OR*: 0.754, 95% *CI*: 0.605–0.940) and increased by 25% on weekends (*OR*: 1.256, 95% *CI*: 1.024–1.542). Among the seven social-ecological dimensions of Model 2, compared with the low-risk group, the high-risk group had an increase in the risk of alcohol consumption by more than 70%. Specifically, at the cultural level, this risk increased by more than three times (*OR*: 3.178, 95% *CI*: 2.405–4.200). Regarding the risk of smoking, the risk increased by more than twice in all six dimensions except the chronosystem (*p* < 0.05).

## 4 Discussion

According to Bronfenbrenner’s ecosystem theory, adolescent health is jointly shaped by interaction environments as well as broader social-cultural contexts including the four levels of micro-, intermediate-, outer- and macro-system (24). Both macro-level conditions and individual personal characteristics jointly drive the emergence of adolescent health-risk behaviors. Consistent with findings from a previous cumulative-risk investigation among Chinese adolescents (16), the research results revealed that each dimension and total score of social ecological risk factors were positively correlated with health risk behaviors. In this study, elevated socio-ecological risk exposure was associated with irregular diet, insufficient physical activity, inadequate sleep, excessive screen time, smoking and alcohol drinking among sampled adolescents, which further supports the value of multi-dimensional socio-ecological assessment for youth behavioral epidemiology.

In addition, this study analyzed the differences in socio-ecological risk factors among adolescents with different demographic characteristics. Demographic differences in health-risk-behavior patterns observed in this dataset further reflect that individual-background factors play a significant role in shaping multiple health risk behaviors in adolescents (25, 26). Parental educational level, family educational ability, and family economy may all have complex impacts on adolescents’ healthy behaviors (26). Gender-specific divergence across multiple socio-ecological dimensions also suggests that environmental risk may operate differently for boys and girls (27).

Participants with urban home addresses, being an only child, attending college, and having more educated parents had higher literacy scores, suggesting that these teenagers were more susceptible to culture shock (13, 28). Such demographic disparities do not act in isolation, they intersect with multi-layer socio-ecological contexts to jointly amplify or buffer behavioral risks (29).

Self-efficacy, as one of the important factors influencing individual behavior, can either indirectly or directly affect the behaviors of children and adolescents, such as unhealthy diet, physical inactivity, smoking and alcohol drinking (30–33). Existing literature supports that self-efficacy constitutes an important internal protective resource for adolescents and higher individual-level protective resources correspond to lower behavioral risk (17). Our findings confirm this view, high individual-dimension risk consistently raises odds for adverse behavioral outcomes.

As the core microsystem for adolescents, adverse family environments including inter-parental conflict, poor parent–child communication and insufficient supervision, may elevate the likelihood of engaging in risk behaviours (34, 35). Parent-adolescent relationships and parental monitoring shape youth smoking and drinking tendencies (36). Beyond direct family influences, peer contexts embedded within family and school also matter. Peer behaviors may drive imitative unhealthy dietary habits, and peer impact grows more salient as adolescents gradually shift away from parental guidance (37–39). Interestingly, the non-significant association between family risk and sleep/physical activity in adjusted models suggests that physical activity and sleep outcomes might be subject to stronger constraints from school-level schedules and peer norms rather than solely family context.

School is the main place for teenagers to study and live outside of home and the school environment is regarded as an important ground to cultivate their behavior (40). The school dimension in this study covers school belonging and peer relationships. Poor sense of school belonging, negative teacher-student interactions and peer relationships jointly contribute to risk behaviours (41, 42). The non-significant for sleep after full adjustment may be partly explained by unified school timetable and homework load, which generate relatively similar sleep conditions for most students (43). School may serve as protective functions even when adolescents face other external adversities (44).

Additionally, elements within the broader community, such as neighborhood characteristics (including social networks and socioeconomic disadvantage), cultural influences, and societal norms, have been shown to significantly shape the development of risk behaviors among youth (45, 46). Our study found that adolescents with higher community risk factors, which reflected the poorer environment of the community, had a higher risk of unhealthy behaviours. Previous studies indicated that community safety can benefit adolescent diet, physical activity, sleep quality and low substance use (smoking and alcohol use) (47–49). The different weekday/weekend screen-time association with community level observed in our study indicated that community environmental functions may produce different behavioural outcomes under distinct daily conditions. Adolescents are mostly constrained by school schedules on weekdays and spend more time in neighbourhood settings on weekends, community disadvantage may reduce access to sports and recreational facilities, pushing adolescents toward sedentary, screen-dominated leisure patterns (50).

At the policy level, from a macro perspective, it is regarded as one of the most important aspects that can influence individual behavior. Previous literature has shown that policies related to schools and communities are associated with negative health behaviors such as poor diet, smoking, and drinking among teenagers (51–53). The results of this study indicate that adolescents with a high risk level in the policy dimension have a higher risk of engaging in various health-risk behaviors including insufficient physical activity, shortened sleep on both weekdays and weekends, prolonged screen time at weekends, smoking and alcohol drinking. This might be due to the lack of support from encouraging policies and prevention from regulatory policies, which have less objective restrictive effects on health-risk behaviors among adolescents (54).

The cultural dimension reflects the degree to which students accept some negative values and things and teenagers with higher scores in the cultural dimension are more likely to develop negative values (40). The cultural ecology of teenagers can provide a buffer for the transitional period of their psychological growth, thereby regulating their behavior and improving their personality, and further influencing a series of their healthy behaviors (14). Cultural-level high-risk status significantly increased odds across nearly all measured health-risk behaviors, and the magnitude of effect for alcohol consumption stood out compared with most other dimensions.

The chronosystem, reflecting family structural and life-event changes (55), also demonstrated detectable links with part of the health-risk outcomes in this sample. After adjustment, chronosystem level high risk was associated with elevated odds of weekday/weekend long screen-time and alcohol drinking. Previous studies have shown that the influence of the environment on alcohol consumption changes over time. For instance, the family or community environment has a greater impact on substance use during early adolescence, while the peer or school environment has a more significant effect during the later stage of adolescence (55).

The study has its limitations. Firstly, the cross-sectional design limited causal inference, and subsequent studies will benefit from a longitudinal design that can identify the determinants of health risk behaviors in adolescents. Then, self-reported questionnaires probably resulted in questions being misinterpreted or acquiescence bias presumably occurred, especially among teenagers who possibly answered questions about health risk behaviors hesitantly. Some behavioral indicators rely on single-item measurement, which may restrict measurement validity. Third, participants were recruited via convenience sampling from several schools in Zhengzhou. Findings cannot be directly generalized to all Chinese adolescents. Further research needs to use multi-item measurement scales and expand sampling coverage across China. Despite the above limitations, the current research has several strengths. First, the research sample used in this study is quite large, providing a basis for the study of risky behaviors related to the social ecology of the Chinese youth population. Second, this study covers seven dimensions from a social ecology perspective and eight types of health-risk behaviors, rather than merely conducting analysis based on a single dimension.

## 5 Conclusion

Each dimension of social ecological risk factors was correlated with health risk behaviors. Findings from this study underline the necessity of multi-level integrated intervention strategies for adolescent health risk behavior prevention. Intervention cannot merely focus on individual level health education. Protective resources should be built across individual self-efficacy promotion, positive family interaction, supportive school climate, accessible community facilities, youth-oriented policy formulation and positive cultural guidance.

## Data Availability

The data analyzed in this study is subject to the following licenses/restrictions: privacy or ethical restrictions. Requests to access these datasets should be directed to wangxian@zzu.edu.cn.
