# Supplementary material for: Socio-ecological risk factors associated with different health-risk behaviors among Chinese adolescents: a cross-sectional study
Source: Front Public Health. 2026 Oct 27;14:1735957. doi: 10.3389/fpubh.2026.1735957 (PMC13762738; doi:10.3389/fpubh.2026.1735957)
Supplement: Supplementary file 1 [file Table_1.DOCX]

**Table A1: The results of the sensitivity analysis**

|  | **Original data** | **Final data** | **χ^2^** | ***P*** |
| --- | --- | --- | --- | --- |
| **Sex** |  |  | 0.091 | 0.763 |
| Male | 1646(43.7) | 2124(56.3) |  |  |
| Female | 1731(44.0) | 2203(56.0) |  |  |
| **Family residence** |  |  | 0.212 | 0.645 |
| Rural | 1984(44.6) | 2463(55.4) |  |  |
| Urban | 1393(44.1) | 1767(55.9) |  |  |
| **Only child** |  |  | 0.117 | 0.732 |
| Yes | 414(44.9) | 509(55.1) |  |  |
| No | 2963(44.3) | 3732(55.7) |  |  |
| **Father’s education level** |  |  | 0.311 | 0.958 |
| Primary or below | 615(43.8) | 789(56.2) |  |  |
| Junior high school | 1539(44.0) | 1959(56.0) |  |  |
| Senior high school | 757(43.3) | 991(56.7) |  |  |
| Junior college or above | 466(44.3) | 587(55.7) |  |  |
| **Mother’s education level** |  |  | 0.102 | 0.992 |
| Primary or below | 895(44.0) | 1139(56.0) |  |  |
| Junior high school | 1380(43.7) | 1778(56.3) |  |  |
| Senior high school | 697(43.6) | 903(56.4) |  |  |
| Junior college or above | 405(44.0) | 515(56.0) |  |  |
| **Self-reported family income** |  |  | 1.635 | 0.442 |
| Under- moderate | 635(44.9) | 778(55.1) |  |  |
| Moderate | 2405(43.7) | 3104(56.3) |  |  |
| Over moderate | 337(42.2) | 462(57.8) |  |  |
| **Close friends** |  |  | 1.242 | 0.743 |
| 0 | 62(40.8) | 90(59.2) |  |  |
| 1-2 | 920(44.0) | 1171(56.0) |  |  |
| 3-5 | 1633(44.3) | 2052(55.7) |  |  |
| >5 | 762(43.2) | 1003(56.8) |  |  |
| **Self-reported study burden** |  |  | 0.124 | 0.940 |
| Under- moderate | 169(44.4) | 212(55.6) |  |  |
| Moderate | 2037(43.9) | 2599(56.1) |  |  |
| Over moderate | 1171(43.6) | 1515(56.4) |  |  |

**Table A2: The results of the internal consistency analysis of the questionnaire**

| **The dimension** | **The questionnaire items** | **Cronbach's Alpha/**  **KMO** | **The correlation with**  **the total score** | **The correlation**  **with its dimension** | **Cronbach's Alpha**  **of its dimension** |
| --- | --- | --- | --- | --- | --- |
|  |  |  |  |  |  |
| **Individual level** | 1.I can always solve problems if I try my best. + | 0.893/  0.908 | 0.448^**^ | 0.615^**^ | 0.711 |
|  | 2.I can stick to some things very well, such as physical exercise and extracurricular reading. + |  | 0.416^**^ | 0.605^**^ |  |
|  | 3.I am confident that I can deal effectively with anything that comes my way. + |  | 0.442^**^ | 0.671^**^ |  |
|  | 4.I feel like I can’t accomplish a lot of things. ++ |  | 0.394^**^ | 0.521^**^ |  |
|  | 5.When encountering setbacks, I can relieve myself. + |  | 0.437^**^ | 0.625^**^ |  |
|  | 6.When encountering painful things, I can use positive behaviors to alleviate the pain. + |  | 0.515^**^ | 0.679^**^ |  |
|  | 7.I like to keep unhappy things to myself and not show them, but I can't forget them. ++ |  | 0.365^**^ | 0.495^**^ |  |
|  | 8.When encountering unhappy things, I tend to vent anger on others and often lose temper. ++ |  | 0.348^**^ | 0.475^**^ |  |
|  |  |  |  |  |  |
| **Family level** | 9.My parents and I can share our inner thoughts well. + |  | 0.487^**^ | 0.732^**^ | 0.816 |
|  | 10.My parents respect my feelings. + |  | 0.501^**^ | 0.781^**^ |  |
|  | 11.My parents support my healthy behaviors. + |  | 0.442^**^ | 0.567^**^ |  |
|  | 12.My parents know when I'm upset about something. + |  | 0.493^**^ | 0.688^**^ |  |
|  | 13.My family members get along well with each other. + |  | 0.505^**^ | 0.708^**^ |  |
|  | 14.Many of my troubles are unknown to my parents. ++ |  | 0.418^**^ | 0.590^**^ |  |
|  | 15.My parents often quarrel over my affairs. ++ |  | 0.361^**^ | 0.572^**^ |  |
|  | 16.My parents often scold me for the slightest thing. ++ |  | 0.413^**^ | 0.669^**^ |  |
|  |  |  |  |  |  |
| **School level** | 17.My teacher likes me very much. + |  | 0.527^**^ | 0.636^**^ | 0.864 |
|  | 18.There are many things I like at school. + |  | 0.615^**^ | 0.738^**^ |  |
|  | 19.I think I can fit in the school life well. + |  | 0.636^**^ | 0.744^**^ |  |
|  | 20.I get along well with my classmates. + |  | 0.575^**^ | 0.674^**^ |  |
|  | 21.When some classmates bully the weak, there will always be someone around me to stop them. + |  | 0.495^**^ | 0.593^**^ |  |
|  | 22.I often study together with my classmates. + |  | 0.522^**^ | 0.616^**^ |  |
|  | 23.I think the teacher is fair to every student. + |  | 0.562^**^ | 0.680^**^ |  |
|  | 24.I feel very uncomfortable at school. ++ |  | 0.579^**^ | 0.649^**^ |  |
|  | 25.I think the teacher’s attitude towards me often depends on my grades in the exams. ++ |  | 0.472^**^ | 0.573^**^ |  |
|  | 26.What the teacher taught was boring. ++ |  | 0.567^**^ | 0.625^**^ |  |
|  | 27.I don’t think the standard of education in schools is good. ++ |  | 0.518^**^ | 0.582^**^ |  |
|  | 28.I was attacked by my classmates' gossip at school. ++ |  | 0.471^**^ | 0.498^**^ |  |
|  |  |  |  |  |  |
| **Community** | 29.There is often an unpleasant smell in the air of the place where one lives (including the dormitory). ++ |  | 0.456^**^ | 0.674^**^ | 0.689 |
|  | 30.The place where I live (including the dormitory) is relatively close to the road, and the noise affects my rest. ++ |  | 0.432^**^ | 0.669^**^ |  |
|  | 31.There is no dedicated place for exercise around the place where one lives (including the dormitory). ++ |  | 0.362^**^ | 0.713^**^ |  |
|  | 32.It is inconvenient to see a doctor at the place where one lives (including the dormitory). ++ |  | 0.420^**^ | 0.757^**^ |  |
|  | 33.There are strangers around where I live (including the dormitory) making dangerous moves towards me. ++ |  | 0.382^**^ | 0.521^**^ |  |
|  |  |  |  |  |  |
| **Public policy** | 34.The school has a reward policy for students who participate in the sports meeting. + |  | 0.279^**^ | 0.537^**^ | 0.718 |
|  | 35.Schools have punitive measures for intentional damage to public property. + |  | 0.168^**^ | 0.465^**^ |  |
|  | 36.School meals are relatively hygienic. + |  | 0.503^**^ | 0.650^**^ |  |
|  | 37.The community encourages everyone to participate in community activities. + |  | 0.529^**^ | 0.691^**^ |  |
|  | 38.The school strictly supervises the Internet cafes and entertainment venues around it. + |  | 0.457^**^ | 0.763^**^ |  |
|  | 39.The community strictly supervises the surrounding Internet cafes and entertainment venues. + |  | 0.441^**^ | 0.740^**^ |  |
|  |  |  |  |  |  |
| **Culture level** | 45.I think the things my idol does (such as tattoos) are acceptable to me. ++ |  | 0.214^**^ | 0.581^**^ | 0.608 |
|  | 46.I think it's better to stay at home and play live streaming or Douyin than to go out. ++ |  | 0.363^**^ | 0.545^**^ |  |
|  | 47.I’ll buy anything expensive as long as I like it. ++ |  | 0.271^**^ | 0.560^**^ |  |
|  | 48.I can accept plastic surgery. ** |  | 0.242^**^ | 0.649^**^ |  |
|  | 49.I think chatting on mobile phones is more fun than chatting in person, for example, we can send emoticons. ++ |  | 0.309^**^ | 0.591^**^ |  |
|  | 50.I think premarital sex is ok as long as two people are mutually agreeable. ++ |  | 0.277^**^ | 0.578^**^ |  |
|  |  |  |  |  |  |
| **Chronosystem** | 40.My parents got divorced in the past three years. + |  | 0.135^**^ | 0.504^**^ | 0.419 |
|  | 41.Family members have been sentenced for criminal or other crimes in the past three years. + |  | 0.167^**^ | 0.383^**^ |  |
|  | 42.In the past three years, my parents have raised a younger brother or sister. + |  | 0.095^**^ | 0.511^**^ |  |
|  | 43.I have been living in new places for the past three years. + |  | 0.162^**^ | 0.711^**^ |  |
|  | 44.A family member has suffered from a serious illness in the past three years. + |  | 0.215^**^ | 0.613^**^ |  |

Note: + means positive factor, ++ means negative factor, ** means *P* < 0.001

**Table A3**: **Comparison of socio-ecological risk scores among different demographic characteristics**

| **Variables** | **N (%)** | **Individual level** | **Family level** | **School level** | **Community level** | **Public policy** | **Culture level** | **Chronosystem** | **Total risk score** |
| --- | --- | --- | --- | --- | --- | --- | --- | --- | --- |
| **Gender** |  |  |  |  |  |  |  |  |  |
| Male | 1646(48.7) | 19.64±4.44 | 18.28±5.31 | 27.84±7.74 | 10.99±3.98 | 14.01±4.59 | 12.27±4.07 | 6.82±2.75 | 109.85±21.77 |
| Female | 1731(51.3) | 20.75±4.45 | 17.71±5.52 | 27.34±7.17 | 9.93±3.37 | 13.50±4.14 | 13.42±4.17 | 6.91±2.68 | 109.56±20.14 |
| ***P*** |  | <0.001 | 0.002 | 0.054 | <0.001 | <0.001 | <0.001 | 0.328 | 0.684 |
| **Only child status** |  |  |  |  |  |  |  |  |  |
| Yes | 414(12.3) | 19.39±4.68 | 17.70±5.76 | 26.40±7.38 | 9.43±3.43 | 12.94±4.18 | 13.41±4.36 | 6.91±2.76 | 106.17±21.24 |
| No | 2963(87.7) | 20.32±4.44 | 18.03±5.38 | 27.75±7.45 | 10.59±3.74 | 13.86±4.39 | 12.78±4.13 | 6.86±2.71 | 110.19±20.86 |
| ***P*** |  | <0.001 | 0.271 | <0.001 | <0.001 | <0.001 | 0.004 | 0.749 | <0.001 |
| **Residential areas** |  |  |  |  |  |  |  |  |  |
| Rural | 1984(58.8) | 20.58±4.24 | 18.05±5.06 | 28.40±7.17 | 11.11±3.60 | 14.21±4.21 | 12.68±4.00 | 6.73±2.59 | 111.77±19.58 |
| Urban | 1393(41.2) | 19.68±4.75 | 17.89±5.91 | 26.42±7.69 | 9.50±3.68 | 13.08±4.51 | 13.11±4.37 | 7.06±2.87 | 106.75±22.44 |
| ***P*** |  | <0.001 | 0.421 | <0.001 | <0.001 | <0.001 | 0.004 | <0.001 | <0.001 |
| **Learning period** |  |  |  |  |  |  |  |  |  |
| Junior high school | 915(27.1) | 19.78±4.83 | 18.42±6.19 | 25.62±8.16 | 9.25±3.73 | 12.63±4.68 | 11.44±4.12 | 7.06±2.90 | 104.21±23.27 |
| Senior high school | 931(27.6) | 20.74±4.52 | 18.01±5.23 | 29.23±7.53 | 11.20±3.74 | 15.09±4.45 | 13.28±4.06 | 6.80±2.61 | 114.35±20.94 |
| Vocational high school | 781(23.1) | 20.48±4.14 | 18.52±5.11 | 28.71±7.14 | 11.57±3.53 | 13.73±4.22 | 12.58±3.89 | 6.61±2.63 | 112.20±19.15 |
| University | 750(22.2) | 19.79±4.24 | 16.87±4.80 | 26.76±5.97 | 9.80±3.29 | 13.45±3.54 | 14.36±4.02 | 6.98±2.67 | 108.00±17.82 |
| ***P*** |  | <0.001 | <0.001 | <0.001 | <0.001 | <0.001 | <0.001 | 0.004 | <0.001 |
| **Father’s education** |  |  |  |  |  |  |  |  |  |
| Primary or below | 615(18.2) | 20.67±4.41 | 18.74±5.42 | 28.71±7.32 | 10.97±3.72 | 14.27±4.18 | 12.55±4.01 | 6.82±2.62 | 112.72±19.75 |
| Junior high school | 1539(45.6) | 20.35±4.35 | 18.02±5.20 | 27.59±7.41 | 10.64±3.69 | 13.82±4.29 | 12.65±4.02 | 6.84±2.69 | 109.91±20.71 |
| Senior high school | 757(22..4) | 20.04±4.50 | 17.87±5.58 | 27.36±7.65 | 10.14±3.81 | 13.60±4.68 | 12.93±4.26 | 6.86±2.69 | 108.80±21.55 |
| Junior college or above | 466(13.8) | 19.38±4.85 | 17.06±5.77 | 26.44±7.29 | 9.64±3.51 | 13.05±4.27 | 13.86±4.51 | 7.05±2.93 | 106.47±21.75 |
| ***P*** |  | <0.001 | <0.001 | <0.001 | <0.001 | <0.001 | <0.001 | 0.465 | <0.001 |
| **Mother’s education** |  |  |  |  |  |  |  |  |  |
| Primary or below | 895(26.5) | 20.64±4.33 | 18.55±5.17 | 28.48±7.17 | 10.92±3.59 | 14.25±4.26 | 12.68±3.88 | 6.79±2.46 | 112.31±19.47 |
| Junior high school | 1380(40.9) | 20.37±4.33 | 18.16±5.36 | 27.53±7.40 | 10.51±3.70 | 13.77±4.33 | 12.66±4.08 | 6.73±2.63 | 109.81±20.81 |
| Senior high school | 697(20.6) | 19.00±4.63 | 17.40±5.56 | 27.26±7.67 | 10.12±3.88 | 13.63±4.53 | 12.97±4.41 | 7.09±2.98 | 108.36±21.82 |
| Junior college or above | 405(12.0) | 19.22±4.84 | 17.17±5.79 | 26.34±7.69 | 9.74±3.63 | 12.76±4.32 | 13.75±4.47 | 7.12±2.98 | 106.10±22.30 |
| ***P*** |  | <0.001 | <0.001 | <0.001 | <0.001 | <0.001 | <0.001 | 0.006 | <0.001 |
| **Self-perceived family economy** |  |  |  |  |  |  |  |  |  |
| Under- moderate | 635(18.8) | 21.23±4.48 | 19.42±5.70 | 29.34±7.24 | 11.20±3.71 | 14.61±4.18 | 13.02±4.05 | 7.35±2.76 | 116.17±19.98 |
| Moderate | 2405(71.2) | 20.18±4.30 | 17.80±5.20 | 27.43±7.26 | 10.38±3.63 | 13.73±4.33 | 12.83±4.13 | 6.71±2.65 | 109.07±20.29 |
| Over moderate | 337(10.0) | 18.45±5.16 | 16.58±5.93 | 25.36±8.45 | 9.51±4.11 | 12.24±4.59 | 12.79±4.57 | 7.06±2.91 | 102.00±23.84 |
| ***P*** |  | <0.001 | <0.001 | <0.001 | <0.001 | <0.001 | 0.549 | <0.001 | <0.001 |
| **The number of close friends** |  |  |  |  |  |  |  |  |  |
| 0 | 62(1.8) | 23.40±5.73 | 21.85±6.35 | 35.58±9.30 | 12.82±4.43 | 15.42±5.29 | 13.23±5.83 | 7.55±3.37 | 129.85±25.67 |
| 1-2 | 920(27.2) | 21.69±4.44 | 19.32±5.41 | 29.58±7.48 | 10.69±3.74 | 14.16±4.31 | 13.03±4.24 | 7.06±2.81 | 115.52±20.56 |
| 3-5 | 1633(48.4) | 19.97±4.18 | 17.58±5.16 | 27.27±6.95 | 10.33±3.57 | 13.74±4.19 | 12.81±4.00 | 6.87±2.71 | 108.57±19.66 |
| >5 | 762(22.6) | 18.66±4.38 | 16.94±5.47 | 25.19±7.26 | 10.22±3.87 | 13.13±4.65 | 12.72±4.25 | 6.58±2.51 | 103.45±20.93 |
| ***P*** |  | <0.001 | <0.001 | <0.001 | <0.001 | <0.001 | 0.372 | <0.001 | <0.001 |
| **Self-perceived study burden** |  |  |  |  |  |  |  |  |  |
| Under- moderate | 169(5.0) | 17.62±5.01 | 16.32±5.68 | 25.02±8.59 | 9.89±4.28 | 12.87±5.05 | 12.57±4.74 | 6.99±3.07 | 101.28±27.14 |
| Moderate | 2037(60.3) | 19.69±4.19 | 17.58±5.17 | 26.57±6.98 | 10.16±3.47 | 13.50±4.24 | 12.54±3.98 | 6.68±2.56 | 106.72±19.54 |
| Over moderate | 1171(34.7) | 21.48±4.55 | 18.93±5.67 | 29.71±7.61 | 11.03±3.97 | 14.31±4.43 | 13.46±4.32 | 7.18±2.89 | 116.09±20.71 |
| ***P*** |  | <0.001 | <0.001 | <0.001 | <0.001 | <0.001 | <0.001 | <0.001 | <0.001 |

**Table A4: Comparison of health risk behaviors with different demographic characteristics**

| **Variables** | **Diet** | | |  | **Physical exercise** | | |  | **Weekday sleep duration** | | |  | **Weekend sleep duration** | | |  | **Weekday screen time** | | |  | **Weekend screen time** | | |  | **Drinking** | | |  | **Smoking** | | |
| --- | --- | --- | --- | --- | --- | --- | --- | --- | --- | --- | --- | --- | --- | --- | --- | --- | --- | --- | --- | --- | --- | --- | --- | --- | --- | --- | --- | --- | --- | --- | --- |
|  | **Healthy** |  | **Unhealthy** |  | **Healthy** |  | **Unhealthy** |  | **Healthy** |  | **Unhealthy** |  | **Healthy** |  | **Unhealthy** |  | **Healthy** |  | **Unhealthy** |  | **Healthy** |  | **Unhealthy** |  | **Healthy** |  | **Unhealthy** |  | **Healthy** |  | **Unhealthy** |
| **Gender** |  |  |  |  |  |  |  |  |  |  |  |  |  |  |  |  |  |  |  |  |  |  |  |  |  |  |  |  |  |  |  |
| Male | 1199(72.8) |  | 447(27.2) |  | 688(41.8) |  | 958(58.2) |  | 234(14.2) |  | 1412(85.8) |  | 911(55.3) |  | 735(44.7) |  | 1126(68.4) |  | 520(31.6) |  | 546(33.2) |  | 1100(66.8) |  | 1293(78.6) |  | 353(21.4) |  | 1475(89.6) |  | 171(10.4) |
| Female | 1232(71.2) |  | 499(28.8) |  | 266(15.4) |  | 1465(84.6) |  | 174(10.1) |  | 1557(89.9) |  | 1068(61.7) |  | 663(38.3) |  | 1226(70.8) |  | 505(29.2) |  | 553(31.9) |  | 1178(68.1) |  | 1599(92.4) |  | 132(7.6) |  | 1703(98.4) |  | 28(1.6) |
| ***P*** | 0.280 |  |  |  | <0.001 |  |  |  | <0.001 |  |  |  | <0.001 |  |  |  | 0.127 |  |  |  | 0.448 |  |  |  | <0.001 |  |  |  | <0.001 |  |  |
| **Only child status** |  |  |  |  |  |  |  |  |  |  |  |  |  |  |  |  |  |  |  |  |  |  |  |  |  |  |  |  |  |  |  |
| Yes | 288(69.6) |  | 126(30.4) |  | 143(34.5) |  | 271(65.5) |  | 29(7.0) |  | 385(93.0) |  | 240(58.0) |  | 174(42.0) |  | 255(61.6) |  | 159(38.4) |  | 139(33.6) |  | 275(66.4) |  | 355(85.7) |  | 59(14.3) |  | 399(96.4) |  | 15(3.6) |
| No | 2143(72.3) |  | 820(27.7) |  | 811(27.4) |  | 2152(72.6) |  | 379(12.8) |  | 2584(87.2) |  | 1739(58.7) |  | 1224(41.3) |  | 2097(70.8) |  | 866(29.2) |  | 960(32.4) |  | 2003(67.6) |  | 2537(85.6) |  | 426(14.4) |  | 2779(93.8) |  | 184(6.2) |
| ***P*** | 0.241 |  |  |  | 0.002 |  |  |  | <0.001 |  |  |  | 0.781 |  |  |  | <0.001 |  |  |  | 0.633 |  |  |  | 0.945 |  |  |  | 0.036 |  |  |
| **Residential areas** |  |  |  |  |  |  |  |  |  |  |  |  |  |  |  |  |  |  |  |  |  |  |  |  |  |  |  |  |  |  |  |
| Rural | 1404(70.8) |  | 580(29.2) |  | 424(21.4) |  | 1560(78.6) |  | 318(16.0) |  | 1666(84.0) |  | 1177(59.3) |  | 807(40.7) |  | 1312(66.1) |  | 672(33.9) |  | 581(29.3) |  | 1403(70.7) |  | 1682(84.8) |  | 302(15.2) |  | 1836(92.5) |  | 148(7.5) |
| Urban | 1027(73.7) |  | 366(26.3) |  | 530(38.0) |  | 863(62.0) |  | 90(6.5) |  | 1303(93.5) |  | 802(57.6) |  | 591(42.4) |  | 1040(74.7) |  | 353(25.3) |  | 518(37.2) |  | 875(62.8) |  | 1210(86.9) |  | 183(13.1) |  | 1342(96.3) |  | 51(3.7) |
| ***P*** | 0.059 |  |  |  | <0.001 |  |  |  | <0.001 |  |  |  | 0.309 |  |  |  | <0.001 |  |  |  | <0.001 |  |  |  | 0.089 |  |  |  | <0.001 |  |  |
| **Learning period** |  |  |  |  |  |  |  |  |  |  |  |  |  |  |  |  |  |  |  |  |  |  |  |  |  |  |  |  |  |  |  |
| Junior high school | 687(75.1) |  | 228(24.9) |  | 331(36.2) |  | 584(63.8) |  | 33(3.6) |  | 882(96.4) |  | 536(58.6) |  | 379(41.4) |  | 744(81.3) |  | 171(18.7) |  | 402(43.9) |  | 513(56.1) |  | 818(89.4) |  | 97(10.6) |  | 882(96.4) |  | 33(3.6) |
| Senior high school | 705(75.7) |  | 226(24.3) |  | 255(27.4) |  | 676(72.6) |  | 10(1.1) |  | 921(98.9) |  | 434(46.6) |  | 497(53.4) |  | 878(94.3) |  | 53(5.7) |  | 360(38.7) |  | 571(61.3) |  | 810(87.0) |  | 121(13.0) |  | 896(96.2) |  | 35(3.8) |
| Vocational high school | 575(73.6) |  | 206(26.4) |  | 219(28.0) |  | 562(72.0) |  | 259(33.2) |  | 522(66.8) |  | 642(82.2) |  | 139(17.8) |  | 504(64.5) |  | 277(35.5) |  | 181(23.2) |  | 600(76.8) |  | 613(78.5) |  | 168(21.5) |  | 669(85.7) |  | 112(14.3) |
| University | 464(61.9) |  | 286(38.1) |  | 149(19.9) |  | 601(80.1) |  | 106(14.1) |  | 644(85.9) |  | 367(48.9) |  | 383(51.1) |  | 226(30.1) |  | 524(69.9) |  | 156(20.8) |  | 594(79.2) |  | 651(86.8) |  | 99(13.2) |  | 731(97.5) |  | 19(2.5) |
| ***P*** | <0.001 |  |  |  | <0.001 |  |  |  | <0.001 |  |  |  | <0.001 |  |  |  | <0.001 |  |  |  | <0.001 |  |  |  | <0.001 |  |  |  | <0.001 |  |  |
| **Father’s education** |  |  |  |  |  |  |  |  |  |  |  |  |  |  |  |  |  |  |  |  |  |  |  |  |  |  |  |  |  |  |  |
| Primary or below | 438(71.2) |  | 177(28.8) |  | 132(21.5) |  | 483(78.5) |  | 101(16.4) |  | 514(83.6) |  | 339(55.1) |  | 276(44.9) |  | 405(65.9) |  | 210(34.1) |  | 177(28.8) |  | 438(71.2) |  | 517(84.1) |  | 98(15.9) |  | 569(92.5) |  | 46(7.5) |
| Junior high school | 1108(72.0) |  | 431(28.0) |  | 413(26.8) |  | 1126(73.2) |  | 209(13.6) |  | 1330(86.4) |  | 939(61.0) |  | 600(39.0) |  | 1100(71.5) |  | 439(28.5) |  | 499(32.4) |  | 1040(67.6) |  | 1313(85.3) |  | 226(14.7) |  | 1431(93.0) |  | 108(7.0) |
| Senior high school | 554(73.2) |  | 203(26.8) |  | 247(32.6) |  | 510(67.4) |  | 72(9.5) |  | 685(90.5) |  | 440(58.1) |  | 317(41.9) |  | 528(69.7) |  | 229(30.3) |  | 256(33.8) |  | 501(66.2) |  | 664(87.7) |  | 93(12.3) |  | 723(95.5) |  | 34(4.5) |
| Junior college or above | 331(71.0) |  | 135(29.0) |  | 162(34.8) |  | 304(65.2) |  | 26(5.6) |  | 440(94.4) |  | 261(56.0) |  | 205(44.0) |  | 319(68.5) |  | 147(31.5) |  | 167(35.8) |  | 299(64.2) |  | 398(85.4) |  | 68(14.6) |  | 455(97.6) |  | 11(2.4) |
| ***P*** | 0.818 |  |  |  | <0.001 |  |  |  | <0.001 |  |  |  | 0.044 |  |  |  | 0.074 |  |  |  | 0.077 |  |  |  | 0.257 |  |  |  | <0.001 |  |  |
| **Mother’s education** |  |  |  |  |  |  |  |  |  |  |  |  |  |  |  |  |  |  |  |  |  |  |  |  |  |  |  |  |  |  |  |
| Primary or below | 622(69.5) |  | 273(30.5) |  | 187(20.9) |  | 708(79.1) |  | 156(17.4) |  | 739(82.6) |  | 522(58.3) |  | 373(41.7) |  | 567(63.4) |  | 328(36.6) |  | 239(26.7) |  | 656(73.3) |  | 753(84.1) |  | 142(15.9) |  | 824(92.1) |  | 71(7.9) |
| Junior high school | 1014(73.5) |  | 366(26.5) |  | 403(29.2) |  | 977(70.8) |  | 158(11.4) |  | 1222(88.6) |  | 821(59.5) |  | 559(40.5) |  | 1012(73.3) |  | 368(26.7) |  | 462(33.5) |  | 918(66.5) |  | 1197(86.7) |  | 183(13.3) |  | 1299(94.1) |  | 81(5.9) |
| Senior high school | 504(72.3) |  | 193(27.7) |  | 226(32.4) |  | 471(67.6) |  | 73(10.5) |  | 624(89.5) |  | 403(57.8) |  | 294(42.2) |  | 496(71.2) |  | 201(28.8) |  | 249(35.7) |  | 448(64.3) |  | 593(85.1) |  | 104(14.9) |  | 659(94.5) |  | 38(5.5) |
| Junior college or above | 291(71.9) |  | 114(28.1) |  | 138(34.1) |  | 267(65.9) |  | 21(5.2) |  | 384(94.8) |  | 233(57.5) |  | 172(42.5) |  | 277(68.4) |  | 128(31.6) |  | 149(36.8) |  | 256(63.2) |  | 349(86.2) |  | 56(13.8) |  | 396(97.8) |  | 9(2.2) |
| ***P*** | 0.230 |  |  |  | <0.001 |  |  |  | <0.001 |  |  |  | 0.838 |  |  |  | <0.001 |  |  |  | <0.001 |  |  |  | 0.351 |  |  |  | <0.001 |  |  |
| **Self-perceived family economy** |  |  |  |  |  |  |  |  |  |  |  |  |  |  |  |  |  |  |  |  |  |  |  |  |  |  |  |  |  |  |  |
| Under moderate | 427(67.2) |  | 208(32.8) |  | 156(24.6) |  | 479(75.4) |  | 87(13.7) |  | 548(86.3) |  | 356(56.1) |  | 279(43.9) |  | 357(56.2) |  | 278(43.8) |  | 167(26.3) |  | 468(73.7) |  | 517(81.4) |  | 118(18.6) |  | 592(93.2) |  | 43(6.8) |
| Moderate | 1756(73.0) |  | 649(27.0) |  | 664(27.6) |  | 1741(72.4) |  | 298(12.4) |  | 2107(87.6) |  | 1435(59.7) |  | 970(40.3) |  | 1735(72.1) |  | 670(27.9) |  | 792(32.9) |  | 1613(67.1) |  | 2096(87.2) |  | 309(12.8) |  | 2264(94.1) |  | 141(5.9) |
| Over moderate | 248(73.6) |  | 89(26.4) |  | 134(39.8) |  | 203(60.2) |  | 23(6.8) |  | 314(93.2) |  | 188(55.8) |  | 149(44.2) |  | 260(77.2) |  | 77(22.8) |  | 140(41.5) |  | 197(58.5) |  | 279(82.8) |  | 58(17.2) |  | 322(95.5) |  | 15(4.5) |
| ***P*** | 0.012 |  |  |  | <0.001 |  |  |  | 0.005 |  |  |  | 0.141 |  |  |  | <0.001 |  |  |  | <0.001 |  |  |  | <0.001 |  |  |  | 0.341 |  |  |
| **The number of close friends** |  |  |  |  |  |  |  |  |  |  |  |  |  |  |  |  |  |  |  |  |  |  |  |  |  |  |  |  |  |  |  |
| 0 | 40(64.5) |  | 22(35.5) |  | 17(27.4) |  | 45(72.6) |  | 6(9.7) |  | 56(90.3) |  | 34(54.8) |  | 28(45.2) |  | 47(75.8) |  | 15(24.2) |  | 27(43.5) |  | 35(56.5) |  | 51(82.3) |  | 11(17.7) |  | 55(88.7) |  | 7(11.3) |
| 1-2 | 640(69.6) |  | 280(30.4) |  | 207(22.5) |  | 713(77.5) |  | 91(9.9) |  | 829(90.1) |  | 535(58.2) |  | 385(41.8) |  | 647(70.3) |  | 273(29.7) |  | 311(33.8) |  | 609(66.2) |  | 807(87.7) |  | 113(12.3) |  | 893(97.1) |  | 27(2.9) |
| 3-5 | 1179(72.2) |  | 454(27.8) |  | 433(26.5) |  | 1200(73.5) |  | 207(12.7) |  | 1426(87.3) |  | 942(57.7) |  | 691(42.3) |  | 1135(69.5) |  | 498(30.5) |  | 510(31.2) |  | 1123(68.8) |  | 1422(87.1) |  | 211(12.9) |  | 1542(94.4) |  | 91(5.6) |
| >5 | 572(75.1) |  | 190(24.9) |  | 297(39.0) |  | 465(61.0) |  | 104(13.6) |  | 658(86.4) |  | 468(61.4) |  | 294(38.6) |  | 523(68.6) |  | 239(31.4) |  | 251(32.9) |  | 511(67.1) |  | 612(80.3) |  | 150(19.7) |  | 688(90.3) |  | 74(9.7) |
| ***P*** | 0.046 |  |  |  | <0.001 |  |  |  | 0.079 |  |  |  | 0.322 |  |  |  | 0.637 |  |  |  | 0.143 |  |  |  | <0.001 |  |  |  | <0.001 |  |  |
| **Self-perceived study burden** |  |  |  |  |  |  |  |  |  |  |  |  |  |  |  |  |  |  |  |  |  |  |  |  |  |  |  |  |  |  |  |
| Under moderate | 134(79.3) |  | 35(20.7) |  | 71(40.0) |  | 98(58.0) |  | 31(18.3) |  | 138(81.7) |  | 131(77.5) |  | 38(22.5) |  | 113(66.9) |  | 56(33.1) |  | 64(37.9) |  | 105(62.1) |  | 123(72.8) |  | 46(27.2) |  | 140(82.8) |  | 29(17.2) |
| Moderate | 1493(73.3) |  | 544(26.7) |  | 556(27.3) |  | 1481(72.7) |  | 296(14.5) |  | 1741(85.5) |  | 1213(59.5) |  | 824(40.5) |  | 1399(68.7) |  | 638(31.3) |  | 669(32.8) |  | 1368(67.2) |  | 1764(86.6) |  | 273(13.4) |  | 1920(94.3) |  | 117(5.7) |
| Over moderate | 804(68.7) |  | 367(31.3) |  | 327(27.9) |  | 844(72.1) |  | 81(6.9) |  | 1090(93.1) |  | 635(54.2) |  | 536(45.8) |  | 840(71.7) |  | 331(28.3) |  | 366(31.3) |  | 805(68.7) |  | 1005(85.8) |  | 166(14.2) |  | 1118(95.5) |  | 53(4.5) |
| ***P*** | 0.002 |  |  |  | <0.001 |  |  |  | <0.001 |  |  |  | <0.001 |  |  |  | 0.140 |  |  |  | 0.207 |  |  |  | <0.001 |  |  |  | <0.001 |  |  |

**Table A5: The comparison of health risk behaviors among different socio-ecological risk groupsm**

| **Social-ecological risk** | **N (%)** | **Diet** | | |  | **Physical exercise** | | |  | **Weekday sleep duration** | | |  | **Weekend sleep duration** | | |  | **Weekday screen time** | | |  | **Weekend screen time** | | |  | **Drinking** | | |  | **Smoking** | | |
| --- | --- | --- | --- | --- | --- | --- | --- | --- | --- | --- | --- | --- | --- | --- | --- | --- | --- | --- | --- | --- | --- | --- | --- | --- | --- | --- | --- | --- | --- | --- | --- | --- |
|  |  | **Healthy** |  | **Unhealthy** |  | **Healthy** |  | **Unhealthy** |  | **Healthy** |  | **Unhealthy** |  | **Healthy** |  | **Unhealthy** |  | **Healthy** |  | **Unhealthy** |  | **Healthy** |  | **Unhealthy** |  | **Healthy** |  | **Unhealthy** |  | **Healthy** |  | **Unhealthy** |
| **Individual level** |  |  |  |  |  |  |  |  |  |  |  |  |  |  |  |  |  |  |  |  |  |  |  |  |  |  |  |  |  |  |  |  |
| Low | 875(25.9) | 718(82.1) |  | 157(17.9) |  | 362(41.4) |  | 513(58.6) |  | 113(12.9) |  | 762(87.1) |  | 539(61.6) |  | 336(38.4) |  | 625(71.4) |  | 250(28.6) |  | 349(39.9) |  | 526(60.1) |  | 778(88.9) |  | 97(11.1) |  | 829(94.7) |  | 46(5.3) |
| Moderate | 1752(51.9) | 1238(70.7) |  | 514(29.3) |  | 436(24.9) |  | 1316(75.1) |  | 234(13.4) |  | 1518(86.6) |  | 1038(59.2) |  | 714(40.8) |  | 1207(68.9) |  | 545(31.1) |  | 563(32.1) |  | 1189(67.9) |  | 1500(85.6) |  | 252(14.4) |  | 1646(93.9) |  | 106(6.1) |
| High | 750(22.2) | 475(63.3) |  | 275(36.7) |  | 156(20.8) |  | 594(79.2) |  | 61(8.1) |  | 689(91.9) |  | 402(53.6) |  | 348(46.4) |  | 520(69.3) |  | 230(30.7) |  | 187(24.9) |  | 563(75.1) |  | 614(81.9) |  | 136(18.1) |  | 703(93.7) |  | 47(6.3) |
| ***P*** |  | <0.001 |  |  |  | <0.001 |  |  |  | <0.001 |  |  |  | 0.004 |  |  |  | 0.402 |  |  |  | <0.001 |  |  |  | <0.001 |  |  |  | 0.636 |  |  |
| **Family level** |  |  |  |  |  |  |  |  |  |  |  |  |  |  |  |  |  |  |  |  |  |  |  |  |  |  |  |  |  |  |  |  |
| Low | 942(27.9) | 746(79.2) |  | 196(20.8) |  | 291(30.9) |  | 651(69.1) |  | 114(12.1) |  | 828(87.9) |  | 540(57.3) |  | 402(42.7) |  | 675(71.7) |  | 267(28.3) |  | 370(39.3) |  | 572(60.7) |  | 844(89.6) |  | 98(10.4) |  | 901(95.6) |  | 41(4.4) |
| Moderate | 1597(47.3) | 1147(71.8) |  | 450(28.2) |  | 417(26.1) |  | 1180(73.9) |  | 203(12.7) |  | 1394(87.3) |  | 955(59.8) |  | 642(40.2) |  | 1112(69.6) |  | 485(30.4) |  | 491(30.7) |  | 1106(69.3) |  | 1362(85.3) |  | 235(14.7) |  | 1507(94.4) |  | 90(5.6) |
| High | 838(24.8) | 538(64.2) |  | 300(35.8) |  | 246(29.4) |  | 592(70.6) |  | 91(10.9) |  | 747(89.1) |  | 484(57.8) |  | 354(42.2) |  | 565(67.4) |  | 273(32.6) |  | 238(28.4) |  | 600(71.6) |  | 686(81.9) |  | 152(18.1) |  | 770(91.9) |  | 68(8.1) |
| ***P*** |  | <0.001 |  |  |  | 0.025 |  |  |  | 0.412 |  |  |  | 0.402 |  |  |  | 0.153 |  |  |  | <0.001 |  |  |  | <0.001 |  |  |  | 0.003 |  |  |
| **School level** |  |  |  |  |  |  |  |  |  |  |  |  |  |  |  |  |  |  |  |  |  |  |  |  |  |  |  |  |  |  |  |  |
| Low | 848(25.1) | 703(82.9) |  | 145(17.1) |  | 310(36.6) |  | 538(63.4) |  | 105(12.4) |  | 743(87.6) |  | 533(62.9) |  | 315(37.1) |  | 640(75.5) |  | 208(24.5) |  | 361(42.6) |  | 487(57.4) |  | 764(90.1) |  | 84(9.9) |  | 809(95.4) |  | 39(4.6) |
| Moderate | 1688(50.0) | 1221(72.3) |  | 467(27.7) |  | 442(26.2) |  | 1246(73.8) |  | 211(12.5) |  | 1477(87.5) |  | 979(58.0) |  | 709(42.0) |  | 1121(66.4) |  | 567(33.6) |  | 476(28.2) |  | 1212(71.8) |  | 1466(86.8) |  | 222(13.2) |  | 1606(95.1) |  | 82(4.9) |
| High | 841(24.9) | 507(60.3) |  | 334(39.7) |  | 202(24.0) |  | 639(76.0) |  | 92(10.9) |  | 749(89.1) |  | 467(55.5) |  | 374(44.5) |  | 591(70.3) |  | 250(29.7) |  | 262(31.2) |  | 579(68.8) |  | 662(78.7) |  | 179(21.3) |  | 763(90.7) |  | 78(9.3) |
| ***P*** |  | <0.001 |  |  |  | <0.001 |  |  |  | 0.501 |  |  |  | 0.007 |  |  |  | <0.001 |  |  |  | <0.001 |  |  |  | <0.001 |  |  |  | <0.001 |  |  |
| **Community level** |  |  |  |  |  |  |  |  |  |  |  |  |  |  |  |  |  |  |  |  |  |  |  |  |  |  |  |  |  |  |  |  |
| Low | 1086(32.2) | 853(78.5) |  | 233(21.5) |  | 354(32.6) |  | 732(67.4) |  | 115(10.6) |  | 971(89.4) |  | 660(60.8) |  | 426(39.2) |  | 754(69.4) |  | 332(30.6) |  | 419(38.6) |  | 667(61.4) |  | 987(90.9) |  | 99(9.1) |  | 1050(96.7) |  | 36(3.3) |
| Moderate | 1649(48.8) | 1162(70.5) |  | 487(29.5) |  | 414(25.1) |  | 1235(74.9) |  | 238(14.4) |  | 1411(85.6) |  | 968(58.7) |  | 681(41.3) |  | 1116(67.7) |  | 533(32.3) |  | 466(28.3) |  | 1183(71.7) |  | 1407(85.3) |  | 242(14.7) |  | 1558(94.5) |  | 91(5.5) |
| High | 642(19.0) | 416(64.8) |  | 226(35.2) |  | 186(29.0) |  | 456(71.0) |  | 55(8.6) |  | 587(91.4) |  | 351(54.7) |  | 291(45.3) |  | 482(75.1) |  | 160(24.9) |  | 214(33.3) |  | 428(66.7) |  | 498(77.6) |  | 144(22.4) |  | 570(88.8) |  | 72(11.2) |
| ***P*** |  | <0.001 |  |  |  | <0.001 |  |  |  | <0.001 |  |  |  | 0.045 |  |  |  | 0.002 |  |  |  | <0.001 |  |  |  | <0.001 |  |  |  | <0.001 |  |  |
| **Public policy** |  |  |  |  |  |  |  |  |  |  |  |  |  |  |  |  |  |  |  |  |  |  |  |  |  |  |  |  |  |  |  |  |
| Low | 1028(30.4) | 818(79.6) |  | 210(20.4) |  | 336(32.7) |  | 692(67.3) |  | 139(13.5) |  | 889(86.5) |  | 643(62.5) |  | 385(37.5) |  | 763(74.2) |  | 265(25.8) |  | 427(41.5) |  | 601(58.5) |  | 926(90.1) |  | 102(9.9) |  | 985(95.8) |  | 43(4.2) |
| Moderate | 1659(49.1) | 1168(70.4) |  | 491(29.6) |  | 436(26.3) |  | 1223(73.7) |  | 202(12.2) |  | 1457(87.8) |  | 988(59.6) |  | 671(40.4) |  | 1108(66.8) |  | 551(33.2) |  | 470(28.3) |  | 1189(71.7) |  | 1420(85.6) |  | 239(14.4) |  | 1565(94.3) |  | 94(5.7) |
| High | 690(20.4) | 445(64.5) |  | 245(35.5) |  | 182(26.4) |  | 508(73.6) |  | 67(9.7) |  | 623(90.3) |  | 348(50.4) |  | 342(49.6) |  | 481(69.7) |  | 209(30.3) |  | 202(29.3) |  | 488(70.7) |  | 546(79.1) |  | 144(20.9) |  | 628(91.0) |  | 62(9.0) |
| ***P*** |  | <0.001 |  |  |  | <0.001 |  |  |  | 0.059 |  |  |  | <0.001 |  |  |  | <0.001 |  |  |  | <0.001 |  |  |  | <0.001 |  |  |  | <0.001 |  |  |
| **Culture level** |  |  |  |  |  |  |  |  |  |  |  |  |  |  |  |  |  |  |  |  |  |  |  |  |  |  |  |  |  |  |  |  |
| Low | 1041(30.8) | 851(81.7) |  | 190(18.3) |  | 361(34.7) |  | 680(65.3) |  | 143(13.7) |  | 898(86.3) |  | 644(61.9) |  | 397(38.1) |  | 822(79.0) |  | 219(21.0) |  | 476(45.7) |  | 565(54.3) |  | 937(90.0) |  | 104(10.0) |  | 992(95.3) |  | 49(4.7) |
| Moderate | 1492(44.2) | 1054(70.6) |  | 438(29.4) |  | 397(26.6) |  | 1095(73.4) |  | 183(12.3) |  | 1309(87.7) |  | 848(56.8) |  | 644(43.2) |  | 1026(68.8) |  | 466(31.2) |  | 428(28.7) |  | 1064(71.3) |  | 1294(86.7) |  | 198(13.3) |  | 1409(94.4) |  | 83(5.6) |
| High | 844(25.0) | 526(62.3) |  | 318(37.7) |  | 196(23.2) |  | 648(76.8) |  | 82(9.7) |  | 762(90.3) |  | 487(57.7) |  | 357(42.3) |  | 504(59.7) |  | 340(40.3) |  | 195(23.1) |  | 649(76.9) |  | 661(78.3) |  | 183(21.7) |  | 777(92.1) |  | 67(7.9) |
| ***P*** |  | <0.001 |  |  |  | <0.001 |  |  |  | 0.028 |  |  |  | 0.034 |  |  |  | <0.001 |  |  |  | <0.001 |  |  |  | <0.001 |  |  |  | 0.010 |  |  |
| **Chronosystem** |  |  |  |  |  |  |  |  |  |  |  |  |  |  |  |  |  |  |  |  |  |  |  |  |  |  |  |  |  |  |  |  |
| Low | 1826(54.1) | 1364(74.7) |  | 462(25.3) |  | 535(29.3) |  | 1291(70.7) |  | 244(13.4) |  | 1582(86.6) |  | 1110(60.8) |  | 716(39.2) |  | 1303(71.4) |  | 523(28.6) |  | 620(34.0) |  | 1206(66.0) |  | 1579(86.5) |  | 247(13.5) |  | 1718(94.1) |  | 108(5.9) |
| Moderate | 1124(33.3) | 782(69.6) |  | 342(30.4) |  | 295(26.2) |  | 829(73.8) |  | 118(10.5) |  | 1006(89.5) |  | 640(56.9) |  | 484(43.1) |  | 768(68.3) |  | 356(31.7) |  | 350(31.1) |  | 774(68.9) |  | 968(86.1) |  | 156(13.9) |  | 1062(94.5) |  | 62(5.5) |
| High | 427(12.6) | 285(66.7) |  | 142(33.3) |  | 124(29.0) |  | 303(71.0) |  | 46(10.8) |  | 381(89.2) |  | 229(53.6) |  | 198(46.4) |  | 281(65.8) |  | 146(34.2) |  | 129(30.2) |  | 298(69.8) |  | 345(80.8) |  | 82(19.2) |  | 398(93.2) |  | 29(6.8) |
| ***P*** |  | <0.001 |  |  |  | 0.187 |  |  |  | 0.046 |  |  |  | 0.010 |  |  |  | 0.040 |  |  |  | 0.155 |  |  |  | 0.009 |  |  |  | 0.634 |  |  |
| **Total risk score** |  |  |  |  |  |  |  |  |  |  |  |  |  |  |  |  |  |  |  |  |  |  |  |  |  |  |  |  |  |  |  |  |
| Low | 878(26.0) | 753(85.8) |  | 125(14.2) |  | 331(37.7) |  | 547(62.3) |  | 117(13.3) |  | 761(86.7) |  | 551(62.8) |  | 327(37.2) |  | 666(75.9) |  | 212(24.1) |  | 401(45.7) |  | 477(54.3) |  | 805(91.7) |  | 73(8.3) |  | 845(96.2) |  | 33(3.8) |
| Moderate | 1680(49.7) | 1193(71.0) |  | 487(29.0) |  | 437(26.0) |  | 1243(74.0) |  | 212(12.6) |  | 1486(87.4) |  | 982(58.5) |  | 698(41.5) |  | 1114(66.3) |  | 566(33.7) |  | 466(27.7) |  | 1214(72.3) |  | 1450(86.3) |  | 230(13.7) |  | 1592(94.8) |  | 88(5.2) |
| High | 819(24.3) | 485(59.2) |  | 334(40.8) |  | 186(22.7) |  | 633(77.3) |  | 79(9.6) |  | 740(90.4) |  | 446(54.5) |  | 373(45.5) |  | 572(69.8) |  | 247(30.2) |  | 232(28.3) |  | 587(71.7) |  | 637(77.8) |  | 182(22.2) |  | 741(90.5) |  | 78(9.5) |
| ***P*** |  | <0.001 |  |  |  | <0.001 |  |  |  | 0.043 |  |  |  | 0.002 |  |  |  | <0.001 |  |  |  | <0.001 |  |  |  | <0.001 |  |  |  | <0.001 |  |  |

**Table A6: Logistic analysis between socio-ecological risk and different health risk behaviors**

| **Variables** | **Diet** | | |  |  |  | **Physical exercise** | | |  |  | | **Weekday sleep time** | | |  |  | | **Weekend sleep time** | | | |  |
| --- | --- | --- | --- | --- | --- | --- | --- | --- | --- | --- | --- | --- | --- | --- | --- | --- | --- | --- | --- | --- | --- | --- | --- |
|  | **Model 1** | |  | **Model 2** | |  | **Model 1** | | | **Model 2** | |  | **Model 1** |  | | **Model 2** | |  | **Model 1** | | | **Model 2** | |
|  | ***OR*** | ***(*95%*CI*)** |  | ***OR*** | ***(*95%*CI*)** |  | ***OR*** | ***(*95%*CI*)** |  | ***OR*** | ***(*95%*CI*)** |  | ***OR*** | ***(*95%*CI*)** |  | ***OR*** | ***(*95%*CI*)** |  | ***OR*** | ***(*95%*CI*)** |  | ***OR*** | ***(*95%*CI*)** |
| **Individual level** |  |  |  |  |  |  |  |  |  |  |  |  |  |  |  |  |  |  |  |  |  |  |  |
| Low | 1.000 |  |  | 1.000 |  |  | 1.000 |  |  | 1.000 |  |  | 1.000 |  |  | 1.000 |  |  | 1.000 |  |  | 1.000 |  |
| Moderate | 1.899 | (1.553-2.321) ** |  | 1.836 | (1.495-2.255) ** |  | 2.130 | (1.792-2.531) ** |  | 1.811 | (1.498-2.189) ** |  | 0.962 | (0.756-1.224) |  | 0.972 | (0.749-1.260) |  | 1.103 | (0.934-1.303) |  | 1.087 | (0.916-1.290) |
| High | 2.648 | (2.108-3.325) ** |  | 2.546 | (2.000-3.242) ** |  | 2.687 | (2.152-3.354) ** |  | 1.983 | (1.546-2.544) ** |  | 1.675 | (1.207-2.325) * |  | 1.306 | (0.913-1.866) |  | 1.389 | (1.139-1.693) * |  | 1.313 | (1.063-1.622) * |
| **Family level** |  |  |  |  |  |  |  |  |  |  |  |  |  |  |  |  |  |  |  |  |  |  |  |
| Low | 1.000 |  |  | 1.000 |  |  | 1.000 |  |  | 1.000 |  |  | 1.000 |  |  | 1.000 |  |  | 1.000 |  |  | 1.000 |  |
| Moderate | 1.493 | (1.233-1.808) ** |  | 1.470 | (1.209-1.787) ** |  | 1.265 | (1.059-1.511) * |  | 1.286 | (1.058-1.564) * |  | 0.945 | (0.740-1.208) |  | 1.010 | (0.777-1.314) |  | 0.903 | (0.767-1.063) |  | 0.846 | (0.716-1.000) * |
| High | 2.122 | (1.718-2.622) ** |  | 2.137 | (1.714-2.664) ** |  | 1.067 | (0.850-1.340) |  | 1.141 | (0.918-1.418) |  | 1.130 | (0.843-1.515) |  | 0.976 | (0.710-1.343) |  | 0.982 | (0.814-1.186) |  | 0.866 | (0.711-1.054) |
| **School level** |  |  |  |  |  |  |  |  |  |  |  |  |  |  |  |  |  |  |  |  |  |  |  |
| Low | 1.000 |  |  | 1.000 |  |  | 1.000 |  |  | 1.000 |  |  | 1.000 |  |  | 1.000 |  |  | 1.000 |  |  | 1.000 |  |
| Moderate | 1.854 | (1.506-2.283) ** |  | 1.693 | (1.367-2.097) ** |  | 1.624 | (1.361-1.939) ** |  | 1.363 | (1.118-1.661) * |  | 0.989 | (0.770-1.270) |  | 1.211 | (0.923-1.590) |  | 1.225 | (1.034-1.452) * |  | 1.176 | (0.986-1.403) |
| High | 3.194 | (2.548-4.003) ** |  | 2.991 | (2.360-3.791) ** |  | 1.823 | (1.476-2.251) ** |  | 1.549 | (1.220-1.967) ** |  | 1.151 | (0.854-1.550) |  | 1.272 | (0.918-1.764) |  | 1.355 | (1.115-1.646) * |  | 1.227 | (0.998-1.508) |
| **Community level** |  |  |  |  |  |  |  |  |  |  |  |  |  |  |  |  |  |  |  |  |  |  |  |
| Low | 1.000 |  |  | 1.000 |  |  | 1.000 |  |  | 1.000 |  |  | 1.000 |  |  | 1.000 |  |  | 1.000 |  |  | 1.000 |  |
| Moderate | 1.534 | (1.282-1.836) ** |  | 1.456 | (1.211-1.750) ** |  | 1.443 | (1.219-1.708) ** |  | 1.351 | (1.119-1.631) * |  | 0.702 | (0.554-0.890) * |  | 0.811 | (0.628-1.046) |  | 1.090 | (0.932-1.274) |  | 1.055 | (0.898-1.239) |
| High | 1.989 | (1.600-2.472) ** |  | 1.937 | (1.544-2.431) ** |  | 1.186 | (0.959-1.466) |  | 1.192 | (0.939-1.512) |  | 1.264 | (0.902-1.771) |  | 1.545 | (1.076-2.218) * |  | 1.284 | (1.054-1.565) * |  | 1.171 | (0.953-1.440) |
| **Public policy** |  |  |  |  |  |  |  |  |  |  |  |  |  |  |  |  |  |  |  |  |  |  |  |
| Low | 1.000 |  |  | 1.000 |  |  | 1.000 |  |  | 1.000 |  |  | 1.000 |  |  | 1.000 |  |  | 1.000 |  |  | 1.000 |  |
| Moderate | 1.637 | (1.361-1.970) ** |  | 1.512 | (1.253-1.824) ** |  | 1.362 | (1.149-1.615) ** |  | 1.145 | (0.949-1.381) |  | 1.128 | (0.895-1.422) |  | 1.414 | (1.102-1.814) * |  | 1.134 | (0.967-1.331) |  | 1.127 | (0.957-1.327) |
| High | 2.145 | (1.725-2.666) ** |  | 2.051 | (1.642-2.562) ** |  | 1.355 | (1.095-1.678) * |  | 1.276 | (1.009-1.614) * |  | 1.454 | (1.067-1.980) * |  | 1.616 | (1.162-2.246) * |  | 1.641 | (1.350-1.996) ** |  | 1.557 | (1.274-1.903) ** |
| **Culture level** |  |  |  |  |  |  |  |  |  |  |  |  |  |  |  |  |  |  |  |  |  |  |  |
| Low | 1.000 |  |  | 1.000 |  |  | 1.000 |  |  | 1.000 |  |  | 1.000 |  |  | 1.000 |  |  | 1.000 |  |  | 1.000 |  |
| Moderate | 1.861 | (1.535-2.257) ** |  | 1.777 | (1.461-2.160) ** |  | 1.464 | (1.233-1.739) ** |  | 1.270 | (1.053-1.531) * |  | 1.139 | (0.901-1.440) |  | 1.470 | (1.140-1.896) * |  | 1.232 | (1.048-1.448) * |  | 1.284 | (1.088-1.514) * |
| High | 2.708 | (2.195-3.341) ** |  | 2.505 | (2.015-3.115) ** |  | 1.755 | (1.431-2.153) ** |  | 1.432 | (1.142-1.797) * |  | 1.480 | (1.109-1.974) * |  | 2.034 | (1.484-2.788) ** |  | 1.189 | (0.988-1.431) |  | 1.246 | (1.027-1.513) * |
| **Chronosystem** |  |  |  |  |  |  |  |  |  |  |  |  |  |  |  |  |  |  |  |  |  |  |  |
| Low | 1.000 |  |  | 1.000 |  |  | 1.000 |  |  | 1.000 |  |  | 1.000 |  |  | 1.000 |  |  | 1.000 |  |  | 1.000 |  |
| Moderate | 1.291 | (1.095-1.523) * |  | 1.268 | (1.073-1.499) * |  | 1.165 | (0.986-1.376) |  | 1.127 | (0.941-1.350) |  | 1.315 | (1.041-1.661) * |  | 1.281 | (1.000-1.641) * |  | 1.172 | (1.008-1.363) * |  | 1.151 | (0.988-1.341) |
| High | 1.471 | (1.172-1.846) ** |  | 1.422 | (1.128-1.791) * |  | 1.013 | (0.803-1.277) |  | 0.932 | (0.725-1.199) |  | 1.277 | (0.914-1.785) |  | 1.139 | (0.798-1.627) |  | 1.340 | (1.084-1.657) * |  | 1.297 | (1.046-1.609) * |
| **Total risk score** |  |  |  |  |  |  |  |  |  |  |  |  |  |  |  |  |  |  |  |  |  |  |  |
| Low | 1.000 |  |  | 1.000 |  |  | 1.000 |  |  | 1.000 |  |  | 1.000 |  |  | 1.000 |  |  | 1.000 |  |  | 1.000 |  |
| Moderate | 2.459 | (1.980-3.054) ** |  | 2.308 | (1.849-2.882) ** |  | 1.721 | (1.445-2.050) ** |  | 1.448 | (1.191-1.760) ** |  | 1.065 | (0.836-1.356) |  | 1.285 | (0.986-1.674) |  | 1.198 | (1.013-1.416) * |  | 1.156 | (0.971-1.376) |
| High | 4.148 | (3.279-5.248) ** |  | 4.021 | (3.145-5.141) ** |  | 2.059 | (1.664-2.548) ** |  | 1.779 | (1.400-2.262) ** |  | 1.440 | (1.064-1.949) * |  | 1.475 | (1.059-2.056) * |  | 1.409 | (1.161-1.711) ** |  | 1.287 | (1.048-1.580) * |

**Table A6: (Continued)**

| **Variables** | **Weekday screen time** | | |  |  | | **Weekend screen time** | | |  | |  | | **Drinking** | |  |  | |  | **Smoking** | | |  | |
| --- | --- | --- | --- | --- | --- | --- | --- | --- | --- | --- | --- | --- | --- | --- | --- | --- | --- | --- | --- | --- | --- | --- | --- | --- |
|  | **Model 1** | |  | **Model 2** | |  | **Model 1** | |  | **Model 2** | | |  | **Model 1** | |  | **Model 2** | |  | **Model 1** | |  | **Model 2** | |
|  | ***OR*** | ***(95%CI*)** |  | ***OR*** | ***(95%CI*)** |  | ***OR*** | ***(*95%*CI*)** |  | ***OR*** | ***(*95%*CI*)** | |  | ***OR*** | ***(*95%*CI*)** |  | ***OR*** | ***(*95%*CI*)** |  | ***OR*** | ***(*95%*CI*)** |  | ***OR*** | ***(*95%*CI*)** |
| **Individual level** |  |  |  |  |  |  |  |  |  |  |  | |  |  |  |  |  |  |  |  |  |  |  |  |
| Low | 1.000 |  |  | 1.000 |  |  | 1.000 |  |  | 1.000 |  | |  | 1.000 |  |  | 1.000 |  |  | 1.000 |  |  | 1.000 |  |
| Moderate | 1.129 | (0.945-1.349) |  | 1.136 | (0.926-1.393) |  | 1.401 | (1.184-1.658) ** |  | 1.357 | (1.137-1.621) ** | |  | 1.347 | (1.050-1.730) * |  | 1.596 | (1.229-2.072) ** |  | 1.161 | (0.813-1.657) |  | 1.350 | (0.928-1.964) |
| High | 1.106 | (0.893-1.369) |  | 1.385 | (1.075-1.784) * |  | 1.998 | (1.613-2.474) ** |  | 2.126 | (1.685-2.682) ** | |  | 1.777 | (1.341-2.353) ** |  | 2.792 | (2.048-3.805) ** |  | 1.205 | (0.793-1.831) |  | 2.179 | (1.376-3.451) ** |
| **Family level** |  |  |  |  |  |  |  |  |  |  |  | |  |  |  |  |  |  |  |  |  |  |  |  |
| Low | 1.000 |  |  | 1.000 |  |  | 1.000 |  |  | 1.000 |  | |  | 1.000 |  |  | 1.000 |  |  | 1.000 |  |  | 1.000 |  |
| Moderate | 1.103 | (0.923-1.317) |  | 1.114 | (0.910-1.364) |  | 1.457 | (1.231-1.724) ** |  | 1.448 | (1.215-1.727) ** | |  | 1.486 | (1.156-1.910) * |  | 1.428 | (1.101-1.852) * |  | 1.312 | (0.899-1.916) |  | 1.209 | (0.814-1.795) |
| High | 1.222 | (0.988-1.496) |  | 1.626 | (1.282-2.061) ** |  | 1.631 | (1.336-1.990) ** |  | 1.793 | (1.451-2.217) ** | |  | 1.908 | (1.452-2.508) ** |  | 2.056 | (1.538-2.748) ** |  | 1.941 | (1.302-2.893) * |  | 2.209 | (1.438-3.393) ** |
| **School level** |  |  |  |  |  |  |  |  |  |  |  | |  |  |  |  |  |  |  |  |  |  |  |  |
| Low | 1.000 |  |  | 1.000 |  |  | 1.000 |  |  | 1.000 |  | |  | 1.000 |  |  | 1.000 |  |  | 1.000 |  |  | 1.000 |  |
| Moderate | 1.556 | (1.292-1.875) ** |  | 1.257 | (1.015-1.557) * |  | 1.887 | (1.588-2.243) ** |  | 1.655 | (1.379-1.986) ** | |  | 1.377 | (1.056-1.797) * |  | 1.462 | (1.106-1.932) * |  | 1.059 | (0.717-1.565) |  | 1.116 | (0.739-1.686) |
| High | 1.302 | (1.050-1.614) * |  | 1.234 | (0.962-1.583) |  | 1.638 | (1.342-2.000) ** |  | 1.509 | (1.217-1.872) ** | |  | 2.459 | (1.860-3.252) ** |  | 2.775 | (2.052-3.753) ** |  | 2.121 | (1.426-3.154) ** |  | 2.388 | (1.549-3.683) ** |
| **Community level** |  |  |  |  |  |  |  |  |  |  |  | |  |  |  |  |  |  |  |  |  |  |  |  |
| Low | 1.000 |  |  | 1.000 |  |  | 1.000 |  |  | 1.000 |  | |  | 1.000 |  |  | 1.000 |  |  | 1.000 |  |  | 1.000 |  |
| Moderate | 1.085 | (0.919-1.280) |  | 0.875 | (0.723-1.060) |  | 1.595 | (1.355-1.876) ** |  | 1.408 | (1.187-1.671) ** | |  | 1.715 | (1.339-2.196) ** |  | 1.618 | (1.251-2.092) ** |  | 1.704 | (1.149-2.526) * |  | 1.503 | (0.997-2.266) |
| High | 0.754 | (0.605-0.940) * |  | 0.637 | (0.495-0.820) ** |  | 1.256 | (1.024-1.542) * |  | 1.131 | (0.910-1.407) | |  | 2.883 | (2.184-3.805) ** |  | 2.514 | (1.873-3.373) ** |  | 3.684 | (2.438-5.567) ** |  | 2.777 | (1.789-4.312) ** |
| **Public policy** |  |  |  |  |  |  |  |  |  |  |  | |  |  |  |  |  |  |  |  |  |  |  |  |
| Low | 1.000 |  |  | 1.000 |  |  | 1.000 |  |  | 1.000 |  | |  | 1.000 |  |  | 1.000 |  |  | 1.000 |  |  | 1.000 |  |
| Moderate | 1.432 | (1.204-1.702) ** |  | 1.162 | (0.956-1.413) |  | 1.797 | (1.526-2.117) ** |  | 1.594 | (1.345-1.889) ** | |  | 1.528 | (1.195-1.954) ** |  | 1.531 | (1.186-1.975) * |  | 1.376 | (0.951-1.991) |  | 1.321 | (0.898-1.942) |
| High | 1.251 | (1.010-1.550) * |  | 1.268 | (0.996-1.614) |  | 1.716 | (1.397-2.108) ** |  | 1.612 | (1.301-1.997) ** | |  | 2.394 | (1.819-3.152) ** |  | 2.341 | (1.757-3.119) ** |  | 2.262 | (1.513-3.379) ** |  | 2.094 | (1.373-3.194) ** |
| **Culture level** |  |  |  |  |  |  |  |  |  |  |  | |  |  |  |  |  |  |  |  |  |  |  |  |
| Low | 1.000 |  |  | 1.000 |  |  | 1.000 |  |  | 1.000 |  | |  | 1.000 |  |  | 1.000 |  |  | 1.000 |  |  | 1.000 |  |
| Moderate | 1.705 | (1.417-2.051) ** |  | 1.352 | (1.101-1.659) * |  | 2.094 | (1.775-2.472) ** |  | 1.910 | (1.610-2.265) ** | |  | 1.379 | (1.072-1.773) * |  | 1.502 | (1.158-1.948) * |  | 1.193 | (0.830-1.713) |  | 1.330 | (0.913-1.938) |
| High | 2.532 | (2.067-3.101) ** |  | 1.814 | (1.442-2.282) ** |  | 2.804 | (2.293-3.429) ** |  | 2.449 | (1.983-3.024) ** | |  | 2.494 | (1.922-3.236) ** |  | 3.178 | (2.405-4.200) ** |  | 1.746 | (1.194-2.553) * |  | 2.474 | (1.648-3.715) ** |
| **Chronosystem** |  |  |  |  |  |  |  |  |  |  |  | |  |  |  |  |  |  |  |  |  |  |  |  |
| Low | 1.000 |  |  | 1.000 |  |  | 1.000 |  |  | 1.000 |  | |  | 1.000 |  |  | 1.000 |  |  | 1.000 |  |  | 1.000 |  |
| Moderate | 1.155 | (0.983-1.357) |  | 1.177 | (0.981-1.412) |  | 1.137 | (0.970-1.333) |  | 1.133 | (0.961-1.334) | |  | 1.030 | (0.830-1.278) |  | 1.070 | (0.857-1.336) |  | 0.929 | (0.673-1.281) |  | 1.004 | (0.719-1.400) |
| High | 1.294 | (1.034-1.620) * |  | 1.394 | (1.079-1.800) * |  | 1.188 | (0.945-1.492) |  | 1.209 | (0.955-1.530) | |  | 1.519 | (1.153-2.002) * |  | 1.709 | (1.283-2.278) ** |  | 1.159 | (0.758-1.771) |  | 1.473 | (0.945-2.298) |
| **Total risk score** |  |  |  |  |  |  |  |  |  |  |  | |  |  |  |  |  |  |  |  |  |  |  |  |
| Low | 1.000 |  |  | 1.000 |  |  | 1.000 |  |  | 1.000 |  | |  | 1.000 |  |  | 1.000 |  |  | 1.000 |  |  | 1.000 |  |
| Moderate | 1.596 | (1.327-1.920) ** |  | 1.365 | (1.104-1.687) * |  | 2.190 | (1.847-2.597) ** |  | 2.003 | (1.674-2.398) ** | |  | 1.749 | (1.326-2.308) ** |  | 1.954 | (1.462-2.612) ** |  | 1.415 | (0.941-2.130) |  | 1.596 | (1.038-2.454) * |
| High | 1.357 | (1.094-1.682) * |  | 1.399 | (1.090-1.797) * |  | 2.127 | (1.738-2.603) ** |  | 2.086 | (1.678-2.592) ** | |  | 3.151 | (2.356-4.214) ** |  | 3.964 | (2.894-5.428) ** |  | 2.695 | (1.773-4.097) ** |  | 3.662 | (2.321-5.778) ** |

Note: * means *P*< 0.05, ** means *P*< 0.001;*CI*: Confidence interval; *OR*: Odds ratio.

Model 1: crude model.

Model 2 adjusted for gender, only child status, residential areas, learning period, the education level of father and mother, self-assessed family economy, number of friends and study burden
